# Clinical Phenotype, Treatment Exposure, Treat-to-Target Status, and Longitudinal Outcomes in Juvenile-Onset Systemic Lupus Erythematosus: A Single-Center Retrospective Cohort Study

**DOI:** 10.3390/jcm15208004

**Published:** 2026-10-15

**Authors:** Burcu Bozkaya Yücel, Şeyda Doğantan, Semanur Elmas, Özlem Aydoğ

**Affiliations:** 1 Department of Pediatric Rheumatology, Samsun City Hospital, 55080 Samsun, Türkiye; 2 Department of Pediatric Rheumatology, Başakşehir Çam ve Sakura City Hospital, 34480 Istanbul, Türkiye; 3 Department of Pediatric Rheumatology, Samsun University, 55080 Samsun, Türkiye; 4 Department of Pediatric Nephrology, Acibadem Maslak Hospital, 34457 Istanbul, Türkiye

**Keywords:** juvenile-onset systemic lupus erythematosus, pediatric rheumatology, lupus nephritis, cLLDAS, treat-to-target, treatment outcomes

## Abstract

**Background/Objectives:** Treat-to-target assessment is central to the management of juvenile-onset systemic lupus erythematosus (jSLE). We primarily evaluated last-visit childhood lupus low disease activity state (cLLDAS) status and its limiting components, and secondarily described clinical phenotype, treatment exposure, and outcomes. **Methods:** This retrospective single-center cohort included 54 patients with jSLE followed during 2020–2025; one patient who died 10 days after diagnosis was excluded from last-visit analyses, and the remaining 53 had at least 6 months of follow-up. The Paediatric Rheumatology European Society (PReS)-endorsed cLLDAS definition was applied at the last visit using a retrospectively assigned physician global assessment (PGA). Systemic Lupus Erythematosus Disease Activity Index 2000 (SLEDAI-2K) and laboratory values at diagnosis and the latest assessment were compared descriptively. **Results:** Median age at diagnosis was 15.0 years, and median follow-up was 34.6 months (IQR 21.2–70.6). Biopsy-proven lupus nephritis occurred in 29.6% (class IV, 13/16). Median SLEDAI-2K was 8.0 at diagnosis and 4.0 at the latest assessment. At the last visit, 29/53 patients (54.7%; 95% CI 40.4–68.4) were in cLLDAS, and 32/53 (60.4%) met the SLEDAI-2K and glucocorticoid components; of 24 patients outside the target, 17 had SLEDAI-2K > 4 and 7 exceeded the glucocorticoid ceiling (both in 3). Conservative minimum rates of flare, infection-related hospitalization, and intensive care unit admission were 5.6, 5.2, and 1.4 per 100 patient-years, respectively; one early death occurred. **Conclusions:** Just over half of the patients were in cLLDAS at the last visit, with residual disease activity as the main barrier, followed by glucocorticoid dose. These observational data reflect outcomes within the available follow-up period and cannot establish treatment effectiveness or long-term prognosis.

## 1. Introduction

Systemic lupus erythematosus (SLE) is a chronic autoimmune disease characterized by immune dysregulation, autoantibody production, immune-complex deposition, and multisystem inflammation [1]. Approximately 15–20% of cases begin before adulthood and are classified as juvenile-onset SLE (jSLE) [2]. Compared with adult-onset disease, jSLE is generally associated with greater disease activity, more severe organ involvement, higher treatment requirements, and greater cumulative damage [3,4,5].

Clinical expression is heterogeneous and may involve virtually any organ system. Mucocutaneous, musculoskeletal, hematologic, renal, and neuropsychiatric manifestations are common [4,6,7,8], while lupus nephritis remains a major determinant of long-term morbidity [4,6]. Advances in immunosuppressive therapy and multidisciplinary care have improved survival, and current strategies emphasize early disease control, minimization of glucocorticoid exposure, and sustained remission or low disease activity [9,10,11,12,13]. Nevertheless, flares, infections, treatment toxicity, and organ damage continue to contribute to disease burden [12].

Disease phenotype and outcomes vary across cohorts and populations, supporting the need for contemporary real-world data from different settings [14,15,16]. The primary aim of this study was to describe last-visit childhood lupus low disease activity state (cLLDAS) status, and the components that limited it, in patients with jSLE managed at a tertiary pediatric rheumatology center in Türkiye. Secondary aims were to describe clinical phenotype, cumulative organ involvement, treatment and glucocorticoid exposure, adverse events, differences in disease activity between diagnosis and the latest assessment, and cumulative follow-up outcomes.

## 2. Materials and Methods

### 2.1. Study Design and Patient Selection

This retrospective cohort study was conducted in the Department of Pediatric Rheumatology at Samsun City Hospital, a tertiary referral center in Türkiye. All patients in the departmental jSLE registry who were followed between January 2020 and December 2025 were screened. The diagnosis of jSLE was established clinically by the treating pediatric rheumatologist on the basis of compatible clinical and laboratory features, using the classification criteria current at the time of diagnosis as a guide. Because the cohort included patients who had been diagnosed before 2019 but remained under follow-up during the 2020–2025 study period, the 2019 European Alliance of Associations for Rheumatology/American College of Rheumatology (EULAR/ACR) classification criteria were applied retrospectively for study eligibility and classification purposes only [17]. Eligible patients had symptom onset before 18 years of age and fulfilled the 2019 EULAR/ACR criteria. Because patients are routinely seen at approximately 3-month intervals, a minimum follow-up of 6 months was required to ensure at least two follow-up visits after diagnosis. The cohort included patients diagnosed during the study period (*n* = 22) and earlier-diagnosed patients who remained under follow-up (*n* = 32); for the latter, records from diagnosis onward contributed to cumulative variables.

**Figure 1:**
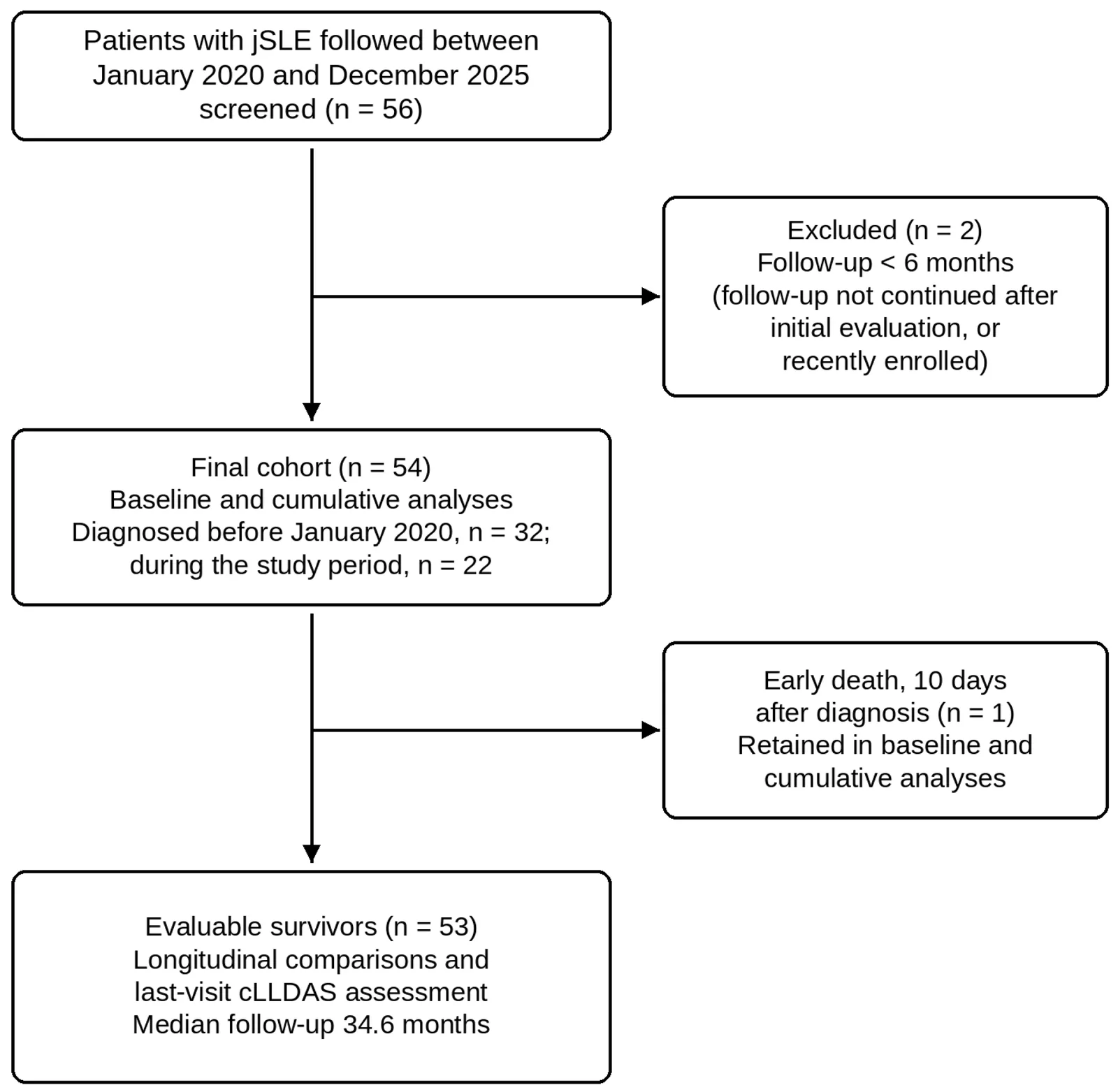
Patient-selection flow diagram. Abbreviations: cLLDAS, childhood lupus low disease activity state; jSLE, juvenile-onset systemic lupus erythematosus.

Of 56 patients identified, two were excluded because follow-up was shorter than 6 months (no follow-up after the initial evaluation or recent enrollment; Figure 1). No patient was transferred to another center or excluded because of incomplete records or failure to fulfill the classification criteria, and no included patient had a gap of more than 6 months in documented follow-up. One patient died 10 days after diagnosis; because death is an outcome rather than incomplete observation, this patient was retained in baseline and cumulative analyses (phenotype, treatment exposure, severe complications, intensive care unit [ICU] admission, and mortality) but excluded from longitudinal and last-visit analyses. The final cohort therefore comprised 54 patients, 53 of whom were evaluable for longitudinal and last-visit analyses; no a priori sample-size calculation was performed. The study followed the Strengthening the Reporting of Observational Studies in Epidemiology (STROBE) statement [18] (Supplementary Table S1).

The study protocol was approved by the Samsun University Non-Interventional Clinical Research Ethics Committee (Decision No: 2026/13/7; 22 July 2026) and was conducted in accordance with the Declaration of Helsinki [19]. Given the retrospective and non-interventional design, the requirement for informed consent was waived.

### 2.2. Data Collection and Definitions

Demographic, clinical, laboratory, treatment, and outcome data were extracted from electronic medical records. Demographic variables included sex, age at symptom onset and diagnosis, diagnostic delay, follow-up duration, and parental consanguinity. Manifestations present at diagnosis were recorded as baseline manifestations, and cumulative manifestations included findings documented from diagnosis through the last follow-up.

Organ involvement was categorized as musculoskeletal, renal, neuropsychiatric, hematologic, and mucocutaneous. Renal involvement was defined as proteinuria attributable to SLE, and lupus nephritis as biopsy-confirmed renal involvement classified according to the revised International Society of Nephrology/Renal Pathology Society classification [20]; biopsy classes were extracted from the original pathology reports. All patients with renal involvement underwent kidney biopsy and had lupus nephritis; no patient with clinically suspected nephritis remained unbiopsied, and no patient had isolated hematuria, active urinary sediment, or SLE-related renal impairment without proteinuria. Neuropsychiatric manifestations included seizure, psychosis, posterior reversible encephalopathy syndrome, chorea, and polyneuropathy; hematologic manifestations included thrombocytopenia, lymphopenia, autoimmune hemolytic anemia, and pancytopenia; and mucocutaneous manifestations included malar rash, oral ulcers, photosensitivity, alopecia, livedoid lesions, panniculitis, and vasculitic skin lesions.

Definite antiphospholipid syndrome (APS) was defined according to the revised Sydney classification criteria [21], and persistent antiphospholipid antibody (aPL) positivity as positivity on at least two occasions at least 12 weeks apart. Macrophage activation syndrome (MAS) was diagnosed by the treating team on the basis of compatible clinical and laboratory features [22].

### 2.3. Laboratory Assessment and Disease Activity

Baseline laboratory variables included hemoglobin, white blood cell count, platelet count, lymphocyte count, erythrocyte sedimentation rate (ESR), C-reactive protein (CRP), albumin, anti-double-stranded DNA antibody (anti-dsDNA), complement component 3 (C3), complement component 4 (C4), and spot urine protein-to-creatinine ratio (UPCR, mg/mg). Anti-dsDNA values > 20 IU/mL were considered positive according to the laboratory reference range; reference ranges for C3 and C4 were 0.90–1.80 g/L and 0.10–0.40 g/L, respectively. Disease activity was assessed with the Systemic Lupus Erythematosus Disease Activity Index 2000 (SLEDAI-2K) [23]. SLEDAI-2K, anti-dsDNA, C3, C4, and UPCR at diagnosis were compared descriptively with values at the latest available assessment; because the interval between assessments varied, this comparison is not a treatment-response analysis. In patients with lupus nephritis, estimated glomerular filtration rate (eGFR) was calculated using the creatinine-based Chronic Kidney Disease in Children Under 25 (CKiD U25) equation [24]; the patient receiving maintenance hemodialysis at the latest assessment was excluded from paired longitudinal analysis because eGFR at that time did not represent native-kidney function. The primary outcome was cLLDAS status at the last visit, assessed in the 53 evaluable patients according to the Paediatric Rheumatology European Society (PReS)-endorsed definition [25]: SLEDAI-2K ≤ 4 with no major organ activity (renal, central nervous system, cardiopulmonary, vasculitis, or fever), no new lupus activity compared with the previous assessment, physician global assessment (PGA) ≤ 1 on a 0–3 scale, prednisolone-equivalent dose ≤ 0.15 mg/kg/day or 7.5 mg/day, whichever is lower, and stable antimalarial, immunosuppressive, and biologic maintenance therapy.

Because PGA had not been recorded prospectively, the last-visit PGA was assigned retrospectively by two treating pediatric rheumatologists, who jointly reviewed the documentation of the last and immediately preceding visits (available for all patients), including clinical findings, laboratory results, and the physician’s narrative assessment, and reached consensus; no predefined scoring rubric was used, and the assessors were not blinded to treatment or outcomes. New lupus activity was assessed by comparing the last visit with the preceding visit (routinely approximately 3 months earlier), including SLEDAI-2K descriptors; any new manifestation documented at the last visit was considered new activity. cLLDAS was adjudicated hierarchically; in patients who already exceeded the SLEDAI-2K or glucocorticoid threshold, the remaining components were not systematically assessed. Because body weight at the last visit was not systematically available, the glucocorticoid criterion was applied using the 7.5 mg/day ceiling. To quantify dependence on the retrospectively assigned PGA, we report the proportion of patients meeting the SLEDAI-2K and glucocorticoid components (SLEDAI-2K ≤ 4 and prednisolone-equivalent dose ≤ 7.5 mg/day) and the number whose cLLDAS status was determined independently of PGA.

### 2.4. Treatment Exposure and Outcomes

Cumulative exposure to hydroxychloroquine, oral corticosteroids, pulse methylprednisolone, cyclophosphamide, mycophenolate mofetil, azathioprine, methotrexate, rituximab, tocilizumab, anakinra, intravenous immunoglobulin (IVIG), plasmapheresis, aspirin, and therapeutic anticoagulation was recorded in the overall cohort; last-visit medications and prednisolone-equivalent doses were summarized among the 53 evaluable patients. All treatment durations refer to treatment actually received. For continuously administered agents, treatment duration was calculated from initiation to discontinuation or the last visit; temporary interruptions were not subtracted. Pulse methylprednisolone and plasmapheresis were summarized as total treatment days. For IVIG, month-based duration was calculated from the first to the last course in patients receiving repeated courses; single doses and short courses were reported separately. For rituximab, exposure was recorded as ever-use, and the planned institutional regimen (375 mg/m^2^ per infusion, two infusions 2 weeks apart, with repeat courses, when given, at 6-month intervals) was distinguished from the treatment actually received. Treatment-associated adverse events were identified from the medical records when the treating physician documented a temporal relationship with the drug that led to dose adjustment, discontinuation, or treatment change. Events were classified as suspected when the documented relationship was possible but uncertain.

Clinical outcomes—disease flare, infection-related hospitalization, ICU admission, and mortality—were assessed cumulatively from diagnosis to the last follow-up visit. Disease flare was defined according to the international consensus definition as a measurable increase in disease activity in one or more organ systems, involving new or worsening clinical signs and symptoms and/or laboratory measurements, considered clinically significant by the treating physician [26]. Flares were identified retrospectively from physician documentation and included, for example, new or worsening arthritis, mucocutaneous lesions, serositis, neuropsychiatric features, cytopenia, or proteinuria. Treatment intensification (an increase in glucocorticoid dose, administration of pulse methylprednisolone, or initiation or change in an immunosuppressive or biologic agent) was considered supportive, but not mandatory, evidence of flare; intensification for refractory disease without an increase in disease activity was not classified as a flare. Infection-related hospitalization was defined as any hospitalization attributable to infection. Outcomes were recorded at the patient level (at least one event); dates and numbers of recurrent events were not systematically available.

### 2.5. Statistical Analysis

Analyses were performed with IBM SPSS Statistics version 21.0 (IBM Corp., Armonk, NY, USA). Because most continuous variables were non-normally distributed on visual and analytical assessment, they are presented as median (interquartile range [IQR]); categorical variables are presented as *n* (%). Available-case analysis was used without imputation; the number of evaluable patients for each analysis is shown in Supplementary Table S2. Values at diagnosis and at the latest assessment for SLEDAI-2K, anti-dsDNA, C3, C4, and UPCR were compared descriptively with the Wilcoxon signed-rank test (paired *n* = 53, 52, 52, 52, and 45, respectively); UPCR was additionally compared in patients with and without lupus nephritis, and eGFR was compared in patients with lupus nephritis, excluding the patient receiving maintenance hemodialysis (*n* = 15). Person-time was calculated from diagnosis to the last recorded follow-up, including pre-2020 follow-up in patients diagnosed before the study period. Because event dates and recurrent events were not systematically recorded, outcome rates were calculated as the number of patients with at least one event per 100 patient-years, with exact Poisson 95% confidence intervals (CIs); these are conservative minimum estimates, and time-to-event analyses were not possible. 95% CIs for proportions were calculated with the Clopper–Pearson exact method; person-time rates and all exact CIs were calculated with Python version 3.13.5 (Python Software Foundation, Wilmington, DE, USA) using the SciPy library version 1.17.0. In a sensitivity analysis, last-visit cLLDAS status and outcome proportions among survivors were stratified by follow-up duration (<24 vs. ≥24 months); the ≥24-month stratum also served as an analysis restricted to patients with at least 2 years of follow-up. Treatment-duration IQRs were calculated using inclusive linear interpolation, and single-patient durations are reported without an IQR or range. Two-sided *p* < 0.05 was considered statistically significant.

During the preparation of this manuscript, the authors used Claude Sonnet 5.5 (Anthropic, San Francisco, CA, USA) solely for language editing. The authors reviewed and edited the output and take full responsibility for the content of this publication.

## 3. Results

### 3.1. Patient Characteristics

Of 56 patients identified, 54 were included (Figure 1; Table 1), of whom 32 (59.3%) were diagnosed before January 2020. Fifty-three patients survived with at least 6 months of follow-up (median 34.6 months, IQR 21.2–70.6; <24 months in 15 [28.3%] and ≥24 months in 38 [71.7%]); one patient died 10 days after diagnosis. Forty-three patients (79.6%) were female, and parental consanguinity was present in 9 (16.7%). Median age at symptom onset was 14.6 years (IQR 12.2–16.0), median age at diagnosis was 15.0 years (IQR 12.6–16.3), and median diagnostic delay was 1.8 months (IQR 0.9–3.3). Antinuclear antibody (ANA) was positive in all patients, and median baseline SLEDAI-2K was 8.0 (IQR 6–12) (Table 1).

### 3.2. Clinical Manifestations

Manifestations at diagnosis and during the disease course are summarized in Table 2. At diagnosis, mucocutaneous and hematologic involvement were present in 29 (53.7%) and 27 (50.0%) patients, respectively; the most frequent individual manifestations were thrombocytopenia (15, 27.8%), arthritis (14, 25.9%), and malar rash (14, 25.9%). Cumulatively, hematologic involvement was the most frequent domain (35/54, 64.8%), followed by mucocutaneous involvement (33/54, 61.1%), biopsy-proven lupus nephritis (16/54, 29.6%), and neuropsychiatric involvement (6/54, 11.1%).

Among the 16 patients with lupus nephritis, 13 (81.3%) had class IV disease, and classes I, II, and V each occurred in one patient; 12 had nephritis at presentation (class IV in 11 and class V in one), and four developed it during follow-up. Persistent aPL positivity was identified in three patients (5.6%), one of whom developed definite APS with arterial thrombosis. MAS occurred in two patients (3.7%), including the early fatal case, and concomitant morphea in two; individual details are provided in Supplementary Table S5.

### 3.3. Treatment Exposure and Adverse Events

Cumulative treatment exposure and duration are summarized in Table 3. Hydroxychloroquine was used in 53 patients (98.1%), oral prednisolone in 46 (85.2%), pulse methylprednisolone in 33 (61.1%), mycophenolate mofetil in 29 (53.7%), IVIG in 21 (38.9%), azathioprine in 19 (35.2%), cyclophosphamide in 15 (27.8%), rituximab in 8 (14.8%), methotrexate in six (11.1%), anakinra in two (3.7%), aspirin in two (3.7%), and therapeutic anticoagulation in one (1.9%). Median treatment duration was 17.5 months (IQR 6.0–31.0; range 0.3–91.0) for oral prednisolone, 34 months (21–77) for hydroxychloroquine, 28 months (19–48) for mycophenolate mofetil, 21 months (10.5–55) for azathioprine, and 23 months (11.1–27) for methotrexate. Pulse methylprednisolone was administered for a median total of 4 days (3–6), cyclophosphamide for 6 months (6–6; range 3–6), and plasmapheresis for 3 days (3–4.5). Among 17 IVIG recipients with month-based records, median duration was 6 months (4–13); three other patients received a single dose and one received a two-day course, and 19 of the 21 IVIG-exposed patients (90.5%) had hematologic, predominantly cytopenic, involvement. Rituximab was used primarily for refractory lupus nephritis or severe systemic disease; two of the eight rituximab-treated patients did not complete the planned regimen because of (suspected) infusion reactions. Tocilizumab, anakinra, and antithrombotic treatment are detailed in Supplementary Table S5. Because cyclophosphamide and rituximab are preferentially used in severe or refractory disease, these patterns are subject to confounding by indication and do not permit inferences about comparative effectiveness.

At the last visit (Table 4), among the 53 evaluable patients, 51 (96.2%) were receiving hydroxychloroquine, 27 (50.9%) oral corticosteroids, 25 (47.2%) mycophenolate mofetil, 12 (22.6%) azathioprine, five (9.4%) methotrexate, two (3.8%) aspirin, one (1.9%) therapeutic anticoagulation, and one (1.9%) tocilizumab. All 53 survivors had received hydroxychloroquine (the early fatal case had not started it); two had discontinued it by the last visit, one because of a visual-field abnormality detected during ophthalmologic screening. The median prednisolone-equivalent dose was 2.5 mg/day (IQR 0–5.0) overall, including patients not receiving corticosteroids, and 5.0 mg/day (IQR 4.0–6.5) among the 27 corticosteroid users. Eight patients (14.8%) had a documented or suspected treatment-associated adverse event, all managed by dose adjustment, discontinuation, or treatment change (Table 4).

### 3.4. Disease Activity and Follow-Up Outcomes

Compared with diagnosis, SLEDAI-2K, anti-dsDNA, and UPCR were lower and C3 was higher at the latest assessment among evaluable survivors (Table 5); because intervals varied, these differences are descriptive and do not represent treatment response. Median SLEDAI-2K was 8.0 (IQR 6–12) at diagnosis and 4.0 (IQR 2–6) at the latest assessment (*p* < 0.001), and median anti-dsDNA was 26.2 (IQR 9.3–62.2) and 14.0 IU/mL (IQR 4.6–26.1), respectively (*p* < 0.001). Median C3 was 0.90 (IQR 0.70–1.10) and 1.10 g/L (IQR 0.90–1.30; *p* < 0.001), whereas C4 did not differ significantly (0.14 [IQR 0.10–0.22] vs. 0.20 g/L [IQR 0.12–0.21]; *p* = 0.109). The overall median UPCR remained 0.10 mg/mg despite a significant paired difference (*p* = 0.001), which reflected the lupus nephritis subgroup: among 15 patients with paired data, median UPCR was 1.78 mg/mg (IQR 0.85–3.15) at diagnosis and 0.11 mg/mg (IQR 0.10–0.45) at the latest assessment (*p* = 0.002); 12 had a reduction of at least 50%, 12 had values ≤0.5 mg/mg, and 8 had values <0.2 mg/mg at the latest assessment, and 10 of the 13 with UPCR >0.5 mg/mg at diagnosis reached ≤0.5 mg/mg. Among 30 patients without lupus nephritis, median UPCR was 0.10 mg/mg at both timepoints (*p* = 0.586), and none had UPCR >0.5 mg/mg at either timepoint.

At the latest pediatric assessment, one of the 16 patients with lupus nephritis was receiving maintenance hemodialysis. Another patient underwent kidney transplantation after the end of pediatric follow-up; this subsequent event was reported separately and was not counted as an outcome within the observation period. Excluding the patient receiving hemodialysis, median eGFR in the remaining 15 patients was 82.3 mL/min/1.73 m^2^ (IQR 51.4–99.6) at diagnosis and 119.8 mL/min/1.73 m^2^ (IQR 111.8–153.7) at the latest assessment (*p* = 0.018); of six patients with serum creatinine >0.9 mg/dL at diagnosis, five had values below 0.9 mg/dL at the latest assessment.

Treat-to-target status and follow-up outcomes are summarized in Table 6. At the last visit, 29 of 53 evaluable patients (54.7%; 95% CI 40.4–68.4) were in cLLDAS, whereas 24 (45.3%) were outside the target state. Twenty-one patients were outside cLLDAS based on the SLEDAI-2K and/or glucocorticoid criteria: 17 had SLEDAI-2K > 4 and 7 had a prednisolone-equivalent dose >7.5 mg/day, with both criteria present in 3 patients. Among the remaining 32 patients (60.4%) who met both criteria, 29 fulfilled cLLDAS. Of the other three, two had a PGA of 2 (one of whom also had major organ activity, new lupus activity, and a change in maintenance therapy, whereas the other failed only the PGA criterion), and one had major organ (renal) activity (Supplementary Table S3). Overall, cLLDAS status could be determined independently of PGA in 23 patients, whereas PGA contributed to the final classification in 30. Among the 17 patients with SLEDAI-2K > 4, anti-dsDNA was positive in 8/16 and complement was low in 4/16, whereas UPCR exceeded 0.5 mg/mg in only 1/16; all had at least one clinical SLEDAI-2K descriptor.

Cumulative clinical outcomes were assessed in the full cohort of 54 patients over 213.4 patient-years. Because outcomes were recorded at the patient level, conservative minimum rates were 5.6 per 100 patient-years (95% CI 2.9–9.8) for disease flare (12/54, 22.2%), 5.2 per 100 patient-years (95% CI 2.6–9.2) for infection-related hospitalization (11/54, 20.4%), and 1.4 per 100 patient-years (95% CI 0.3–4.1) for ICU admission (3/54, 5.6%). Ten of the 12 patients with a flare underwent treatment intensification, whereas treatment was intensified without a flare in four patients because of refractory disease. One flare occurred after patient-initiated discontinuation of maintenance treatment. One early death (1/54, 1.9%) occurred 10 days after diagnosis in a patient with newly diagnosed jSLE complicated by pulmonary hemorrhage and macrophage activation syndrome, followed by multiorgan failure.

In the sensitivity analysis stratified by follow-up duration, cLLDAS was present in 7/15 (46.7%) survivors with <24 months of follow-up and in 22/38 (57.9%) with ≥24 months. Flare occurred in 2/15 (13.3%) and 9/38 (23.7%), infection-related hospitalization in 3/15 (20.0%) and 7/38 (18.4%), and ICU admission in 1/15 (6.7%) and 1/38 (2.6%), respectively (Supplementary Table S4).

## 4. Discussion

In this single-center cohort of 54 patients with jSLE, just over half of the evaluable patients (29/53, 54.7%; 95% CI 40.4–68.4) were in cLLDAS at the last visit, with residual disease activity as the main barrier, followed by a glucocorticoid dose above the cLLDAS ceiling. Hematologic and mucocutaneous manifestations predominated, and biopsy-proven lupus nephritis affected 29.6% of patients and was predominantly proliferative. Flare and infection-related hospitalization occurred in 22.2% and 20.4% of patients (at least 5.6 and 5.2 per 100 patient-years, respectively).

The demographic profile was consistent with jSLE cohorts [2,4,6,12]. Hematologic involvement was the most frequent cumulative domain (64.8%), consistent with Turkish multicenter data emphasizing cytopenic manifestations [27], which have been associated with greater overall and steroid-related damage [8], and mucocutaneous involvement (61.1%) was consistent with Turkish and international cohorts [7,12,14,15,16]. Reported musculoskeletal frequencies vary widely, largely depending on whether arthralgia is included [16,28].

Biopsy-proven lupus nephritis occurred in 29.6%, close to the 29.3% reported by Sahin et al. [16]; higher frequencies in other Turkish cohorts partly reflect broader definitions of renal involvement [15]. Because 13 of 16 cases were class IV, the modest overall frequency should not obscure the severity of kidney disease among affected patients. The paired UPCR difference was confined to the nephritis subgroup, in which it was clinically relevant: most patients had a reduction of at least 50% and reached the ≤0.5 mg/mg threshold used in complete renal response definitions [29]. eGFR was also higher at the latest assessment in patients not receiving hemodialysis; these longitudinal differences should not be interpreted as treatment effects, and standardized renal response according to guideline-defined composite criteria could not be assessed retrospectively. Contemporary Kidney Disease: Improving Global Outcomes (KDIGO) and ACR guidelines emphasize rapid control of active nephritis and durable kidney response [29,30], and failure to achieve early remission predicts worse long-term renal outcomes in pediatric cohorts [31,32]; in our cohort, one of the 16 patients was receiving maintenance hemodialysis at the latest pediatric assessment, and another underwent kidney transplantation after the end of pediatric follow-up. Neuropsychiatric involvement (11.1%) was close to the 13.5% reported in a Turkish multicenter study [33].

Persistent antiphospholipid antibody positivity (5.6%), macrophage activation syndrome (3.7%), and exposure to tocilizumab or anakinra each involved very few patients; these observations are descriptive (Supplementary Table S5) and do not permit inferences about treatment effectiveness. The early fatal case, with pulmonary hemorrhage and multiorgan failure despite intensive treatment, underscores the recognized life-threatening potential of MAS in jSLE [22].

Treatment patterns were broadly consistent with the SHARE recommendations available for most of the study period [13] and with current EULAR recommendations [9]: hydroxychloroquine was used in all survivors, mycophenolate mofetil was the most frequent steroid-sparing agent, cyclophosphamide and rituximab were concentrated in severe or refractory disease [29,34,35], and IVIG was used mainly as adjunctive or rescue therapy for severe hematologic manifestations [36,37]. The KDIGO, ACR, and 2026 British Society for Rheumatology (BSR) guidelines [29,30,37] postdate much of the observation period and are cited as current benchmarks rather than as the standards applied during treatment. Last-visit prednisolone-equivalent doses were generally low (median 2.5 mg/day), but oral glucocorticoid exposure lasted a median of 17.5 months, and 7 of the 24 patients outside cLLDAS exceeded the glucocorticoid ceiling; these findings are consistent with recommendations to minimize glucocorticoid exposure whenever disease control permits [9,37,38].

Because last-visit cLLDAS is a cross-sectional status at a single timepoint, it is not directly comparable with studies reporting cLLDAS at any time, at 12 months, or sustained over follow-up: Ozturk et al. reported lupus low disease activity state (LLDAS) at any time in 82% and LLDAS maintained for at least 50% of follow-up (LLDAS-50) in 68.9% [39], Baba et al. 12-month cLLDAS in 50% [38], Bergkamp et al. cLLDAS maintained for at least 50% of follow-up in 58% [40], and Yin et al. cLLDAS at any time in 72.2% but sustained in 30.9% [41]. Our estimate (95% CI 40.4–68.4%) overlaps with these data, but the comparison is indicative only; referral to a tertiary center may also have enriched our cohort for severe disease, as suggested by the frequent use of pulse methylprednisolone (61.1%) and cyclophosphamide (27.8%) and the predominance of proliferative nephritis, which may have lowered the cLLDAS proportion relative to less selected cohorts. Residual disease activity accounted for most patients outside the target, indicating that persistent disease activity, rather than glucocorticoid dose alone, was the principal barrier to cLLDAS in this cohort. Because cLLDAS attainment is associated with lower hazards of subsequent severe flare and damage [42], and the 2026 BSR guideline recommends remission, or cLLDAS when remission is not attainable, as the primary target in childhood SLE [37], prospective recording of cLLDAS components, including PGA, would support treat-to-target implementation and the assessment of sustained target attainment [43]. The differences in SLEDAI-2K, anti-dsDNA, and C3 between diagnosis and the latest assessment describe longitudinal differences rather than treatment response; nevertheless, higher longitudinal disease activity has been associated with damage accrual in Turkish childhood-onset SLE [44].

In a large Medicaid cohort, serious infection incidence was 10.42 per 100 person-years in childhood SLE and 17.65 per 100 person-years with lupus nephritis, with corticosteroid exposure associated with higher risk [45], and infection-related admissions account for a disproportionate share of in-hospital mortality in childhood-onset SLE [46]. Our patient-based rate of infection-related hospitalization (at least 5.2 per 100 patient-years) is a conservative minimum estimate and should be interpreted in this context. The single early death followed severe lupus with pulmonary hemorrhage and MAS rather than infection.

Strengths include detailed cumulative organ phenotyping, treatment exposure and adverse-event data, and a pediatric-specific treat-to-target endpoint with component-level analysis. The study also has important limitations. First, the retrospective single-center design and modest sample size limit statistical power and the precision of the estimates, and findings from small subgroups should be interpreted descriptively. Second, referral to a tertiary center may have enriched the cohort for severe disease and major organ involvement, limiting generalizability to the broader jSLE population. Third, PGA and cLLDAS were adjudicated retrospectively from clinical documentation by treating pediatric rheumatologists without blinding or a predefined PGA scoring rubric, and inter-rater agreement was not assessed; this may have introduced misclassification. Use of the 7.5 mg/day glucocorticoid ceiling may also have overestimated cLLDAS in lighter patients, and because adjudication was hierarchical, failures of components other than SLEDAI-2K and glucocorticoid dose represent minimum estimates. Fourth, follow-up duration was heterogeneous and, for some patients, relatively short for the assessment of long-term outcomes: although the median follow-up was 34.6 months (IQR 21.2–70.6), 15 of the 53 evaluable patients (28.3%) had less than 24 months of observation. Late flares and delayed treatment-related complications may therefore not have been fully captured, and differences in observation time may have influenced cumulative outcome frequencies, because patients with longer follow-up had more opportunity to experience such events; person-time rates only partly account for this. Outcomes that accrue over many years, including organ damage, end-stage kidney disease, and outcomes after transition to adult care, could not be assessed; for example, one patient underwent kidney transplantation after the end of pediatric follow-up. In a sensitivity analysis restricted to patients with at least 24 months of follow-up, the proportion in cLLDAS (57.9%) was similar to that in the overall cohort (Supplementary Table S4); however, the small strata preclude definitive comparisons. In addition, cLLDAS was assessed only at the last visit and reflects disease status at a single timepoint rather than sustained disease control; time to cLLDAS and sustained cLLDAS could not be evaluated. Accordingly, our findings should be interpreted as describing disease status and outcomes within the available follow-up period rather than as estimates of long-term prognosis. Fifth, flares were identified retrospectively from physician documentation rather than with a validated flare instrument; this approach depends on physician judgment and documentation practices, may have introduced misclassification, and limits comparability with studies using validated indices. Sixth, outcomes were recorded at the patient level rather than as recurrent events; therefore, event rates represent conservative minimum estimates, time-to-event analyses were not possible, and infection-related events could not be characterized by pathogen or severity. Seventh, cumulative glucocorticoid dose, standardized renal response according to guideline-defined composite criteria, damage, formal remission, and health-related quality of life were not systematically available. Missing data were also not completely random, particularly for UPCR, which was measured less often in patients without clinically suspected renal involvement (Supplementary Table S2). Finally, treatment exposures were subject to confounding by indication and were interpreted descriptively rather than as comparative effectiveness estimates. Nevertheless, this cohort provides real-world data on treat-to-target status and its barriers in Turkish jSLE.

## 5. Conclusions

In this single-center jSLE cohort from a tertiary referral center, just over half of the evaluable patients were in cLLDAS at the last visit, and residual disease activity was the main barrier, followed by glucocorticoid dose. Biopsy-proven lupus nephritis affected fewer than one-third of patients but was predominantly class IV, and infection-related hospitalization affected one-fifth of the cohort. Given the observational design and variability in follow-up duration, these data describe disease status and outcomes within the available observation period rather than long-term prognosis or sustained disease control, and do not establish treatment effectiveness. These findings highlight the value of prospective recording of cLLDAS components and are consistent with current recommendations for sustained treat-to-target disease control, glucocorticoid minimization, and infection prevention, including vaccination, in jSLE care.

## Figures and Tables

**Table 1:** Demographic, Clinical, and Laboratory Characteristics at Diagnosis.

Variable	Value
Female sex, *n* (%)	43 (79.6)
Consanguinity, *n* (%)	9 (16.7)
Age at symptom onset, years	14.6 (12.2–16.0)
Age at diagnosis, years	15.0 (12.6–16.3)
Diagnostic delay, months	1.8 (0.9–3.3)
Follow-up duration, months	34.6 (21.2–70.6)
Hemoglobin, g/dL	11.4 (9.8–12.7)
White blood cell count, ×10^3^/mm^3^	6.8 (4.9–8.9)
Platelet count, ×10^3^/mm^3^	255 (98.5–345.3)
Lymphocyte count, ×10^3^/mm^3^	1.4 (1.1–1.8)
Albumin, g/dL	4.0 (3.1–4.5)
ESR, mm/h	34 (21.5–56.0)
CRP, mg/L	4.5 (3.0–15.0)
Anti-dsDNA, IU/mL	26.2 (9.3–62.2)
C3, g/L	0.90 (0.68–1.10)
C4, g/L	0.13 (0.10–0.22)
Spot urine protein/creatinine ratio, mg/mg	0.10 (0.10–0.65)
Baseline SLEDAI-2K	8.0 (6.0–12.0)

Abbreviations: anti-dsDNA, anti-double-stranded DNA antibody; C3, complement component 3; C4, complement component 4; CRP, C-reactive protein; ESR, erythrocyte sedimentation rate; IQR, interquartile range; SLEDAI-2K, Systemic Lupus Erythematosus Disease Activity Index 2000. Data are presented as median (IQR) unless otherwise specified. Follow-up duration is summarized among the 53 surviving patients with at least 6 months of observation. For baseline anti-dsDNA, *n* = 52; for baseline C3 and C4, *n* = 53; for baseline spot urine protein-to-creatinine ratio, *n* = 47; all other baseline variables in this table had *n* = 54. Reference ranges: anti-dsDNA >20 IU/mL positive; C3 0.90–1.80 g/L; C4 0.10–0.40 g/L.

**Table 2:** Clinical Manifestations at Diagnosis and During the Disease Course.

Domain and Manifestation	At Diagnosis *n* (%)	Cumulative *n* (%)
**Hematologic involvement**	**27 (50.0)**	**35 (64.8)**
Thrombocytopenia	15 (27.8)	15 (27.8)
Lymphopenia	4 (7.4)	14 (25.9)
Autoimmune hemolytic anemia	4 (7.4)	7 (13.0)
Pancytopenia	4 (7.4)	6 (11.1)
**Musculoskeletal involvement**	**14 (25.9)**	**14 (25.9)**
Arthritis	14 (25.9)	14 (25.9)
**Mucocutaneous involvement**	**29 (53.7)**	**33 (61.1)**
Malar rash	14 (25.9)	18 (33.3)
Oral ulcers	10 (18.5)	13 (24.1)
Photosensitivity	2 (3.7)	9 (16.7)
Alopecia	0 (0.0)	3 (5.6)
Livedoid skin lesions	—	2 (3.7)
Panniculitis	—	1 (1.9)
Vasculitic skin manifestations	3 (5.6)	3 (5.6)
**Renal involvement/biopsy-proven lupus nephritis**	**12 (22.2)**	**16 (29.6)**
Class I	0	1 (1.9)
Class II	0	1 (1.9)
Class IV	11 (20.4)	13 (24.1)
Class V	1 (1.9)	1 (1.9)
**Neuropsychiatric involvement**	**4 (7.4)**	**6 (11.1)**
Seizures	1 (1.9)	2 (3.7)
Psychosis	3 (5.6)	3 (5.6)
Posterior reversible encephalopathy syndrome	—	1 (1.9)
Polyneuropathy	—	1 (1.9)
Chorea	1 (1.9)	2 (3.7)
**Other SLE-related manifestations**		
Thrombosis	1 (1.9)	1 (1.9)
Definite antiphospholipid syndrome	—	1 (1.9)
Macrophage activation syndrome	—	2 (3.7)
Pulmonary hemorrhage	—	1 (1.9)
**Concomitant condition**		
Morphea	—	2 (3.7)

Note: At-diagnosis manifestations were those documented at initial presentation, whereas cumulative manifestations included findings recorded at diagnosis or at any time during follow-up. Individual patients could have more than one manifestation within the same clinical domain; therefore, the sum of individual manifestations may exceed the corresponding domain total. Percentages were calculated using the total cohort of 54 patients as the denominator. All patients with renal involvement (proteinuria attributable to SLE) underwent kidney biopsy and had biopsy-proven lupus nephritis; the two definitions therefore comprise the same 16 patients, and the at-diagnosis values refer to renal involvement and biopsy class at presentation. A dash indicates that the value was not applicable. Domain-level totals are shown in bold. Abbreviation: SLE, systemic lupus erythematosus.

**Table 3:** Cumulative Treatment Exposure and Duration During Follow-up.

Treatment (Unit)	Exposure *n* (%)	Duration *n*	Duration Median (IQR)	Min–Max
Hydroxychloroquine (months)	53 (98.1)	53	34.0 (21.0–77.0)	6.0–150.0
Oral prednisolone (months)	46 (85.2)	46	17.5 (6.0–31.0)	0.3–91.0
Pulse methylprednisolone (days)	33 (61.1)	33	4.0 (3.0–6.0)	3.0–18.0
Mycophenolate mofetil (months)	29 (53.7)	29	28.0 (19.0–48.0)	5.0–82.0
Intravenous immunoglobulin (months)	21 (38.9)	17	6.0 (4.0–13.0)	1.0–18.0
Azathioprine (months)	19 (35.2)	19	21.0 (10.5–55.0)	2.0–84.0
Methotrexate (months)	6 (11.1)	6	23.0 (11.1–27.0)	3.0–32.0
Cyclophosphamide (months)	15 (27.8)	15	6.0 (6.0–6.0)	3.0–6.0
Rituximab *	8 (14.8)	—	—	—
Aspirin (months)	2 (3.7)	2	39.2 (37.2–41.1)	35.3–43.0
Therapeutic anticoagulation (months)	1 (1.9)	1	28.9	—
Plasmapheresis (days)	3 (5.6)	3	3.0 (3.0–4.5)	3.0–6.0
Tocilizumab (months)	2 (3.7)	1	8	—
Anakinra (months)	2 (3.7)	1	1.5	—

Note: Exposure percentages use *N* = 54. All durations refer to treatment actually received. Duration *n* denotes treated patients with a numeric duration in the stated unit. Values are presented as median (IQR) and minimum–maximum. Single-patient durations are reported without an IQR or range. Single-dose and short-course exposures were reported separately from month-based duration summaries (Section 3.3; Supplementary Table S5). One patient discontinued cyclophosphamide because of an infusion reaction (Table 4). * No duration was calculated for rituximab. The planned institutional regimen consisted of 375 mg/m^2^ per infusion, planned as two infusions 2 weeks apart, with repeat courses, when given, separated by 6 months; two patients did not complete the planned regimen because of (suspected) infusion reactions (Table 4). Patients could receive more than one treatment. Abbreviations: IQR, interquartile range; IVIG, intravenous immunoglobulin.

**Table 4:** Treatment at Last Follow-up and Treatment-Associated Adverse Events.

**Panel A. Current medications at last follow-up**		
**Medication**		***n*** **(%)**
Hydroxychloroquine		51 (96.2)
Oral corticosteroids		27 (50.9)
Mycophenolate mofetil		25 (47.2)
Azathioprine		12 (22.6)
Methotrexate		5 (9.4)
Aspirin		2 (3.8)
Therapeutic anticoagulation		1 (1.9)
Tocilizumab		1 (1.9)
**Panel B. Treatment-associated adverse events**		
**Adverse event**	***n*** **(%)**	**Clinical management and outcome**
Visual-field abnormality during hydroxychloroquine treatment	1 (1.9)	Hydroxychloroquine was permanently discontinued as a precaution after a visual-field abnormality was detected during ophthalmologic screening.
Azathioprine-associated neutropenia	1 (1.9)	Azathioprine was discontinued because of neutropenia, and immunosuppressive treatment was subsequently continued with mycophenolate mofetil.
Azathioprine-associated gastrointestinal intolerance	1 (1.9)	Gastrointestinal symptoms improved after dose adjustment, allowing continuation of azathioprine treatment without further complications.
Methotrexate-associated gastrointestinal intolerance	1 (1.9)	Methotrexate dose was reduced because of gastrointestinal intolerance, resulting in complete symptom resolution and continuation of therapy.
Rituximab-associated infusion-related reaction	1 (1.9)	Rituximab treatment was discontinued following an infusion-related reaction, and maintenance therapy was continued with azathioprine.
Suspected rituximab-associated infusion reaction	1 (1.9)	Rituximab was discontinued after the first infusion because of a suspected reaction; no subsequent dose or repeat course was administered.
Cyclophosphamide-associated infusion reaction	1 (1.9)	Cyclophosphamide was discontinued because of an infusion reaction, and treatment was subsequently continued with mycophenolate mofetil.
Tocilizumab-associated neutropenia with subsequent infection	1 (1.9)	Tocilizumab was administered off-label as a single intravenous infusion for severe inflammatory musculoskeletal manifestations accompanied by elevated acute-phase reactants. Treatment was discontinued following the development of neutropenia and infection. After resolution of the infection, the patient’s clinical condition improved, and maintenance therapy was continued with mycophenolate mofetil.

Note: Panel A percentages use the 53 patients with evaluable last-visit maintenance treatment; the patient who died 10 days after diagnosis was not included in this denominator. Panel B percentages use the full cohort (*N* = 54). Overall, eight patients (14.8%) had a documented or suspected treatment-associated adverse event. Panel titles and column headings are shown in bold.

**Table 5:** Disease Activity, Serological, and Renal Markers at Diagnosis and at the Latest Assessment.

Variable	Baseline	Latest Assessment	*p* Value
SLEDAI-2K	8.0 (6–12)	4.0 (2–6)	<0.001
Anti-dsDNA, IU/mL	26.2 (9.3–62.2)	14.0 (4.6–26.1)	<0.001
C3, g/L	0.90 (0.70–1.10)	1.10 (0.90–1.30)	<0.001
C4, g/L	0.14 (0.10–0.22)	0.20 (0.12–0.21)	0.109
Spot urine protein/creatinine ratio, mg/mg	0.10 (0.10–0.65)	0.10 (0.10–0.12)	0.001
Lupus nephritis (*n* = 15 paired)	1.78 (0.85–3.15)	0.11 (0.10–0.45)	0.002
No lupus nephritis (*n* = 30 paired)	0.10 (0.08–0.10)	0.10 (0.10–0.10)	0.586
eGFR (CKiD U25), mL/min/1.73 m^2^, lupus nephritis excluding maintenance hemodialysis (*n* = 15 paired)	82.3 (51.4–99.6)	119.8 (111.8–153.7)	0.018

Abbreviations: anti-dsDNA, anti-double-stranded DNA antibody; C3, complement component 3; C4, complement component 4; CKiD U25, Chronic Kidney Disease in Children Under 25 equation; eGFR, estimated glomerular filtration rate; IQR, interquartile range; SLEDAI-2K, Systemic Lupus Erythematosus Disease Activity Index 2000; UPCR, spot urine protein-to-creatinine ratio. Data are presented as median (IQR). The latest assessment refers to the last available visit; because the interval from diagnosis varied between patients, the comparison is descriptive and does not represent treatment response. The patient who died 10 days after diagnosis was excluded from longitudinal comparisons. Available baseline/last-follow-up/paired sample sizes among evaluable survivors were 53/53/53 for SLEDAI-2K, 52/52/52 for anti-dsDNA, 52/52/52 for C3, 52/52/52 for C4, and 47/47/45 for UPCR (15 paired observations in patients with lupus nephritis and 30 in patients without lupus nephritis); eGFR was compared in 15 patients with lupus nephritis, excluding the patient receiving maintenance hemodialysis at the latest assessment. Paired comparisons were performed with the Wilcoxon signed-rank test. Reference ranges: anti-dsDNA >20 IU/mL positive; C3 0.90–1.80 g/L; C4 0.10–0.40 g/L. In patients without lupus nephritis, low UPCR values were frequently recorded as approximately 0.1 mg/mg in the source records; the narrow interquartile range in this group reflects this, and all values were ≤0.20 mg/mg at the latest assessment. Baseline summaries in Table 5 may differ slightly from Table 1 because Table 5 is restricted to evaluable survivors, whereas Table 1 summarizes the overall cohort at diagnosis.

**Table 6:** Treat-to-Target Status at the Last Visit and Cumulative Follow-up Outcomes.

Outcome	*n* (%)
cLLDAS at last follow-up	29 (54.7; 95% CI 40.4–68.4)
Disease flare during follow-up	12 (22.2)
At least one infection-related hospitalization during follow-up	11 (20.4)
ICU admission during follow-up	3 (5.6)
Early death (10 days after diagnosis)	1 (1.9)

Abbreviations: cLLDAS, childhood lupus low disease activity state; ICU, intensive care unit; jSLE, juvenile-onset systemic lupus erythematosus; PReS, Paediatric Rheumatology European Society. cLLDAS was assessed at the last follow-up visit in the 53 surviving patients with at least 6 months of observation using the PReS-endorsed childhood-specific definition; 32/53 (60.4%) met the objectively documented SLEDAI-2K and glucocorticoid components (Supplementary Table S3). All other outcomes in this table use the full cohort (*N* = 54) and represent cumulative events recorded from diagnosis through the last follow-up visit; rates per 100 patient-years are reported in the text and Supplementary Table S4. The patient who died 10 days after diagnosis was retained in cumulative outcome analyses and had newly diagnosed jSLE complicated by pulmonary hemorrhage and macrophage activation syndrome, with subsequent multiorgan failure; this is a cumulative count and not a person-time-based or long-term mortality estimate. Individual patients may have experienced more than one outcome event during follow-up.

## Data Availability

The de-identified data supporting the findings of this study are available from the corresponding author on reasonable request, subject to applicable ethical and privacy restrictions.

## Supplementary Materials

The following supporting information can be downloaded at https://www.mdpi.com/article/10.3390/jcm15208004/s1, Table S1: STROBE checklist; Table S2: Number of evaluable patients and missing data by variable and analysis; Table S3: Components limiting last-visit cLLDAS status; Table S4: Person-time outcome rates and outcomes stratified by follow-up duration; Table S5: Details of uncommon events and treatments.

## Abbreviations

The following abbreviations are used in this manuscript:

ACR	American College of Rheumatology
ANA	antinuclear antibody
anti-dsDNA	anti-double-stranded DNA antibody
aPL	antiphospholipid antibody
APS	antiphospholipid syndrome
BSR	British Society for Rheumatology
C3	complement component 3
C4	complement component 4
CI	confidence interval
CKiD U25	Chronic Kidney Disease in Children Under 25 equation
cLLDAS	childhood lupus low disease activity state
CRP	C-reactive protein
eGFR	estimated glomerular filtration rate
ESR	erythrocyte sedimentation rate
EULAR	European Alliance of Associations for Rheumatology
ICU	intensive care unit
IQR	interquartile range
IVIG	intravenous immunoglobulin
jSLE	juvenile-onset systemic lupus erythematosus
KDIGO	Kidney Disease: Improving Global Outcomes
LLDAS	lupus low disease activity state
LLDAS-50	lupus low disease activity state maintained for at least 50% of follow-up
MAS	macrophage activation syndrome
PGA	physician global assessment
PReS	Paediatric Rheumatology European Society
SLE	systemic lupus erythematosus
SLEDAI-2K	Systemic Lupus Erythematosus Disease Activity Index 2000
STROBE	Strengthening the Reporting of Observational Studies in Epidemiology
UPCR	spot urine protein-to-creatinine ratio
