# Supplementary material for: Clinical Phenotype, Treatment Exposure, Treat-to-Target Status, and Longitudinal Outcomes in Juvenile-Onset Systemic Lupus Erythematosus: A Single-Center Retrospective Cohort Study
Source: J Clin Med. 2026 Oct 15;15(20):8004. doi: 10.3390/jcm15208004 (PMC13705632; doi:10.3390/jcm15208004)
Supplement: Supplementary file 1 [file jcm-15-08004-s001.zip › jcm-4589066-supplementary.pdf]

Supplementary Materials

Clinical Phenotype, Treatment Exposure, Treat-to-Target Status, and Longitudinal Outcomes in Juvenile-Onset Systemic Lupus Erythematosus: A Single-Center Retrospective Cohort Study

Burcu Bozkaya Yücel, Şeyda Doğantan, Semanur Elmas, and Özlem Aydoğ

Contents

Table S1. STROBE checklist

Table S2. Number of evaluable patients and missing data by variable and analysis

Table S3. Components limiting last-visit cLLDAS status

Table S4. Person-time outcome rates and outcomes stratified by follow-up duration

Table S5. Details of uncommon events and treatments

Table S1. STROBE checklist

Item	Recommendation	Reported in
**Title and abstract**		
1a	Indicate the study design with a commonly used term in the title or abstract	Title; Abstract
1b	Provide an informative and balanced summary of what was done and what was found	Abstract
**Introduction**		
2	Background/rationale: explain the scientific background and rationale	Section 1
3	Objectives: state specific objectives	Section 1 (final paragraph)
**Methods**		
4	Study design: present key elements of study design early in the paper	Section 2.1
5	Setting: describe the setting, locations, and relevant dates, including periods of recruitment, follow-up, and data collection	Section 2.1
6a	Participants: eligibility criteria, sources and methods of selection, and methods of follow-up	Section 2.1; Figure 1
6b	Matched studies: matching criteria and number of exposed and unexposed	Not applicable
7	Variables: define outcomes, exposures, and potential confounders; give diagnostic criteria	Sections 2.2–2.4
8	Data sources/measurement: give sources of data and details of assessment methods	Sections 2.2–2.4
9	Bias: describe efforts to address potential sources of bias	Sections 2.3 and 2.5; Section 4 (limitations)
10	Study size: explain how the study size was arrived at	Section 2.1
11	Quantitative variables: explain how quantitative variables were handled in the analyses	Sections 2.3–2.5
12a	Statistical methods, including those used to control for confounding	Section 2.5
12b	Methods used to examine subgroups and interactions	Section 2.5 (lupus nephritis subgroup; follow-up strata)
12c	How missing data were addressed	Section 2.5; Table S2
12d	How loss to follow-up was addressed	Sections 2.1 and 2.5
12e	Sensitivity analyses	Sections 2.3 and 2.5; Tables S3 and S4
**Results**		
13a	Numbers of individuals at each stage of the study	Section 3.1; Figure 1 (56 screened, 2 excluded, 54 included, 53 evaluable)
13b	Reasons for non-participation at each stage	Section 2.1; Section 3.1; Figure 1
13c	Consider use of a flow diagram	Figure 1
14a	Characteristics of study participants	Sections 3.1–3.2; Tables 1 and 2
14b	Number of participants with missing data for each variable of interest	Table 1 and Table 5 footnotes; Table S2

Item	Recommendation	Reported in
14c	Summarize follow-up time	Section 3.1 (median follow-up); Section 3.4 (213.4 patient-years)
15	Outcome data: report numbers of outcome events or summary measures over time	Section 3.4; Table 6
16a	Unadjusted estimates and their precision (e.g., 95% confidence intervals)	Section 3.4; Tables 5 and 6
16b	Report category boundaries when continuous variables were categorized	Section 2.3 (SLEDAI-2K and glucocorticoid thresholds); Section 3.4
16c	Translate estimates of relative risk into absolute risk where relevant	Not applicable; no relative risk estimates were reported
17	Other analyses: subgroups, interactions, and sensitivity analyses	Section 3.4; Table 5; Tables S3 and S4
**Discussion**		
18	Key results with reference to study objectives	Section 4 (first paragraph)
19	Limitations, including sources of potential bias or imprecision	Section 4 (limitations paragraph)
20	Cautious overall interpretation considering objectives, limitations, and other evidence	Sections 4 and 5
21	Generalisability (external validity) of the results	Section 4 (referral setting; limitations)
**Other information**		
22	Funding: source of funding and role of funders	Funding statement

STROBE, Strengthening the Reporting of Observational Studies in Epidemiology. Item descriptions are abbreviated from the STROBE checklist for cohort studies (von Elm et al., 2007).

Table S2. Number of evaluable patients and missing data by variable and analysis

Variable	Baseline, full cohort (N=54)	Baseline, evaluable survivors (n=53)	Last visit, evaluable survivors (n=53)	Paired, evaluable survivors (n=53)
**Disease activity**				
SLEDAI-2K	54	53	53	53
**Serological markers**				
Anti-dsDNA	52	52	52	52
C3	53	52	52	52
C4	53	52	52	52
**Renal markers**				
Spot urine protein/creatinine ratio (UPCR)	47	47	47	45
Lupus nephritis (n=16)	—	—	—	15
No lupus nephritis (n=37)	—	—	—	30
Serum creatinine	54	53	53	53
**Other laboratory variables**				
Hemoglobin, white blood cell, platelet and lymphocyte counts, albumin, ESR, CRP	54	—	—	—
eGFR (CKiD U25), lupus nephritis (n=16)	16	16	15	15
**Treat-to-target and outcome analyses**				
Last-visit cLLDAS	—	—	53	—
Cumulative outcomes (flare, infection-related hospitalization, ICU admission, death)	54	—	—	—

Values are numbers of patients with available data. The patient who died 10 days after diagnosis was included in baseline and cumulative analyses but excluded from longitudinal and last-visit analyses. Paired eGFR analysis excluded the patient receiving maintenance hemodialysis at the latest assessment. Abbreviations: anti-dsDNA, anti-double-stranded DNA antibody; C3, complement component 3; C4, complement component 4; cLLDAS, childhood lupus low disease activity state; CRP, C-reactive protein; CKiD U25, Chronic Kidney Disease in Children Under 25 equation; eGFR, estimated glomerular filtration rate; ESR, erythrocyte sedimentation rate; ICU, intensive care unit; SLEDAI-2K, Systemic Lupus Erythematosus Disease Activity Index 2000; UPCR, spot urine protein-to-creatinine ratio.

Table S2 (continued). Pattern of missing paired UPCR data among evaluable survivors

Characteristic	No paired UPCR (n=8)	Paired UPCR available (n=45)
Lupus nephritis, n	1	15
SLEDAI-2K at diagnosis, median	5.0	8.0
SLEDAI-2K at the latest assessment, median	2.0	4.0

Paired UPCR values were missing mainly in patients without lupus nephritis and with lower disease activity, in whom testing was less often clinically indicated; the data were therefore not missing completely at random.

Table S3. Components limiting last-visit cLLDAS status

cLLDAS component not met	n/53 (%)	n/24 outside cLLDAS (%)	Patients assessed
**Objectively documented components (assessed in all patients)**			
SLEDAI-2K >4	17 (32.1)	17 (70.8)	53
Prednisolone-equivalent dose >7.5 mg/day	7 (13.2)	7 (29.2)	53
Both of the above	3 (5.7)	3 (12.5)	53
At least one of the above	21 (39.6)	21 (87.5)	53
**Remaining components (assessed hierarchically)**			
PGA ≥2	2 (3.8)	2 (8.3)	32
Major organ activity	2 (3.8)	2 (8.3)	32
New lupus activity compared with the preceding assessment	1 (1.9)	1 (4.2)	32
Change in maintenance therapy	1 (1.9)	1 (4.2)	32
PGA as the sole failing criterion	1 (1.9)	1 (4.2)	32
**Summary**			
Met SLEDAI-2K and glucocorticoid components	32 (60.4)	—	53
cLLDAS status determined independently of PGA	23 (43.4)	—	53
PGA required for final classification	30 (56.6)	—	53
In cLLDAS at the last visit	29 (54.7; 95% CI 40.4–68.4)	—	53

Adjudication was hierarchical: in the 21 patients who exceeded the SLEDAI-2K or glucocorticoid threshold, the remaining components were not systematically assessed; frequencies for PGA, major organ activity, new activity, and maintenance-therapy change are therefore minimum estimates. Of the three patients outside cLLDAS among the 32 who met the SLEDAI-2K and glucocorticoid components, two had a PGA of 2 (one of whom also had major organ activity, new disease activity, and a change in maintenance therapy) and one had major organ (renal) activity. The 95% CI was calculated with the Clopper–Pearson exact method. Abbreviations: CI, confidence interval; cLLDAS, childhood lupus low disease activity state; PGA, physician global assessment; SLEDAI-2K, Systemic Lupus Erythematosus Disease Activity Index 2000.

Table S3 (continued). Last-visit laboratory findings among the 17 patients with SLEDAI-2K >4

Finding at the last visit	n/N with available data
Anti-dsDNA positive (>20 IU/mL)	8/16
Low complement (C3 <0.90 g/L or C4 <0.10 g/L)	4/16
UPCR >0.5 mg/mg	1/16
Thrombocytopenia (<100 $\times 10^3/mm^3$) or leukopenia (<3 $\times 10^3/mm^3$)	0/17

Because serological descriptors contribute at most 4 points to SLEDAI-2K, all 17 patients had at least one clinical descriptor contributing to the score. Among the 21 patients outside cLLDAS on objective criteria, UPCR exceeded 0.5 mg/mg in 1 of 19 with available data. Abbreviations as above; UPCR, spot urine protein-to-creatinine ratio.

Table S4. Person-time outcome rates and outcomes stratified by follow-up duration

Panel A. Conservative minimum outcome rates in the full cohort (N=54; 213.4 patient-years)

Outcome	n/54 (%)	Rate per 100 patient-years (95% CI)
Disease flare	12 (22.2)	5.6 (2.9–9.8)
Infection-related hospitalization	11 (20.4)	5.2 (2.6–9.2)
ICU admission	3 (5.6)	1.4 (0.3–4.1)

Total observation time was calculated from diagnosis to the last recorded follow-up for each patient. Because recurrent-event counts and event dates were not systematically available, rates are based on the number of patients with at least one event and represent conservative minimum estimates; 95% CIs are exact Poisson intervals.

Panel B. Outcomes and last-visit cLLDAS status among evaluable survivors stratified by follow-up duration

Variable	Follow-up <24 months (n=15)	Follow-up ≥24 months (n=38)
Follow-up duration, months, median	16.0	44.9
SLEDAI-2K at the last visit, median	2.0	4.0
cLLDAS at the last visit, n (%) [95% CI]	7 (46.7) [21.3–73.4]	22 (57.9) [40.8–73.7]
Disease flare, n (%)	2 (13.3)	9 (23.7)
Infection-related hospitalization, n (%)	3 (20.0)	7 (18.4)
ICU admission, n (%)	1 (6.7)	1 (2.6)

The patient who died 10 days after diagnosis (who had a flare, an infection-related hospitalization, and ICU admission) is excluded from this table and reported separately in the main text. The ≥24-month stratum also represents an analysis restricted to patients with at least 2 years of follow-up. Given the small strata, results are descriptive and were not tested formally; 95% CIs were calculated with the Clopper–Pearson exact method. Abbreviations: CI, confidence interval; cLLDAS, childhood lupus low disease activity state; ICU, intensive care unit; SLEDAI-2K, Systemic Lupus Erythematosus Disease Activity Index 2000.

Table S5. Details of uncommon events and treatments

Condition or exposure	n	Clinical details	Treatment and course
Definite antiphospholipid syndrome	1	Distal popliteal artery thrombosis with thrombotic toe involvement	Therapeutic anticoagulation from diagnosis throughout follow-up (28.9 months); on anticoagulation at the last visit
Persistent antiphospholipid antibody positivity without definite APS	2	Persistent positivity on at least two occasions ≥12 weeks apart	Aspirin throughout follow-up (35.3 and 43.0 months); both on aspirin at the last visit
Livedoid lesions	2	One patient had chorea with livedoid lesions; another had recurrent livedoid lesions with persistent antiphospholipid antibody positivity	—
Macrophage activation syndrome	1	jSLE-associated MAS	Pulse methylprednisolone followed by anakinra for 1.5 months
Macrophage activation syndrome (early fatal case)	1	Newly diagnosed jSLE complicated by MAS and pulmonary hemorrhage	Pulse methylprednisolone, plasmapheresis, and a 5-day course of anakinra; multiorgan failure and death 10 days after diagnosis
Concomitant morphea	2	One patient also had biopsy-confirmed celiac disease	In one patient with persistent morphea activity, subcutaneous tocilizumab for 8 months after methotrexate, hydroxychloroquine, and monthly IVIG
Tocilizumab, single intravenous dose	1	Severe inflammatory musculoskeletal manifestations accompanied by elevated acute-phase reactants	Administered off-label as a single intravenous infusion. Treatment was discontinued following the development of neutropenia and infection. After resolution of the infection, the patient's clinical condition improved, and maintenance therapy was continued with mycophenolate mofetil

These exposures are reported descriptively and should not be interpreted as evidence of treatment efficacy. Definite antiphospholipid syndrome was defined according to the revised Sydney classification criteria; MAS was diagnosed by the treating team. Abbreviations: APS, antiphospholipid syndrome; IVIG, intravenous immunoglobulin; jSLE, juvenile-onset systemic lupus erythematosus; MAS, macrophage activation syndrome.
